# Functional Diversification of Oyster Big Defensins Generates Antimicrobial Specificity and Synergy against Members of the Microbiota

**DOI:** 10.3390/md20120745

**Published:** 2022-11-26

**Authors:** Noémie De San Nicolas, Aromal Asokan, Rafael D. Rosa, Sébastien N. Voisin, Marie-Agnès Travers, Gustavo Rocha, Luc Dantan, Yann Dorant, Guillaume Mitta, Bruno Petton, Guillaume M. Charrière, Jean-Michel Escoubas, Viviane Boulo, Juliette Pouzadoux, Hervé Meudal, Karine Loth, Vincent Aucagne, Agnès F. Delmas, Philippe Bulet, Caroline Montagnani, Delphine Destoumieux-Garzón

**Affiliations:** 1 IHPE, University Montpellier, CNRS, Ifremer, University Perpignan Via Domitia, 34090 Montpellier, France; 2 Centre de Biophysique Moléculaire UPR4301 CNRS, 45071 Orléans, France; 3 Laboratory of Immunology Applied to Aquaculture, Department of Cell Biology, Embryology and Genetics, Federal University of Santa Catarina, Florianópolis 88040-900, SC, Brazil; 4 Plateforme BioPark d’Archamps, Archparc, 74160 Archamps, France; 5 Ifremer, IRD, ILM, Université de Polynésie Française, UMR EIO, Vairao 98179, French Polynesia; 6 Ifremer, CNRS, IRD, Ifremer, LEMAR UMR 6539, Université de Bretagne Occidentale, 29840 Argenton-en-Landunvez, France; 7 UFR ST, Université d’Orléans, 45067 Orléans, France; 8 CR UGA, IAB, INSERM U1209, CNRS UMR5309, 74160 La Tronche-Archamps, France

**Keywords:** antibacterial peptide, beta defensin, diversity, evolution, microbiome, invertebrate, mollusk

## Abstract

Big defensins are two-domain antimicrobial peptides (AMPs) that have highly diversified in mollusks. *Cg*-BigDefs are expressed by immune cells in the oyster *Crassostrea gigas*, and their expression is dampened during the Pacific Oyster Mortality Syndrome (POMS), which evolves toward fatal bacteremia. We evaluated whether *Cg*-BigDefs contribute to the control of oyster-associated microbial communities. Two *Cg*-BigDefs that are representative of molecular diversity within the peptide family, namely *Cg*-BigDef1 and *Cg*-BigDef5, were characterized by gene cloning and synthesized by solid-phase peptide synthesis and native chemical ligation. Synthetic peptides were tested for antibacterial activity against a collection of culturable bacteria belonging to the oyster microbiota, characterized by 16S sequencing and MALDI Biotyping. We first tested the potential of *Cg*-BigDefs to control the oyster microbiota by injecting synthetic *Cg*-BigDef1 into oyster tissues and analyzing microbiota dynamics over 24 h by 16S metabarcoding. *Cg*-BigDef1 induced a significant shift in oyster microbiota β-diversity after 6 h and 24 h, prompting us to investigate antimicrobial activities in vitro against members of the oyster microbiota. Both *Cg*-BigDef1 and *Cg*-BigDef5 were active at a high salt concentration (400 mM NaCl) and showed broad spectra of activity against bacteria associated with *C. gigas* pathologies. Antimicrobial specificity was observed for both molecules at an intra- and inter-genera level. Remarkably, antimicrobial spectra of *Cg*-BigDef1 and *Cg*-BigDef5 were complementary, and peptides acted synergistically. Overall, we found that primary sequence diversification of *Cg*-BigDefs has generated specificity and synergy and extended the spectrum of activity of this peptide family.

## 1. Introduction

From invertebrates to humans, active crosstalk between the host immune system and the microbiota plays a critical role in maintaining homeostasis [1,2]. Antimicrobial peptides (AMPs), among other immune effectors, are key players in host-microbe interactions [3,4,5,6]. AMPs encompass a highly diverse array of molecules widespread in multicellular organisms, which were initially described for their direct antimicrobial activities against pathogens [7,8]. AMPs are multifunctional: they are involved in the early establishment and shaping of bacterial microbiota; they maintain tolerance to beneficial microbes and greatly affect community composition in the guts, epithelia, and mucosal surfaces of mammals through direct and indirect activities against commensal bacteria [9,10]. In other animal branches as well, AMPs play an important role in host-microbiota interactions [5,11]. In arthropods such as insects and crustaceans, AMPs regulate microbiota composition [6,12,13]. In cnidarians, they are crucial in shaping microbial colonization during *Hydra* development [14].

Several families of AMPs have been identified in *Crassostrea gigas* oysters (mollusks) and characterized in terms of expression, structure, and function [15,16,17]. AMP families in oysters have widely diversified; they are expressed at low concentrations by immune cells, hemocytes, and epithelia [15]. Recent studies have highlighted the role of host-microbiota interactions in oyster health [18,19]. The structure of the oyster microbiota is modified under stressful conditions promoting the development of opportunistic infections [18]. The resulting dysbiosis can be associated with significant mortality. In particular, *C. gigas* suffers from a polymicrobial disease called the Pacific Oyster Mortality Syndrome (POMS), which is triggered by infection with the OsHV-1 µVar virus and affects the oyster’s immune cells. Interestingly, hemocyte infection has been associated with attenuation of AMP expression with a loss of barrier function leading to dysbiosis and fatal bacteremia [20,21]. Oyster big defensins are among the peptide families whose expression is altered during POMS, suggesting that they may contribute to the control of oyster microbial communities [21]. This hypothesis is further supported by the recent finding that a big defensin mediates microbial shaping in another bivalve mollusk, the scallop *Argopecten purpuratus* [22].

Knowledge of big defensins has significantly increased over the past decade, particularly with the growing availability of next-generation sequencing data. Phylogenetic analyses have shown that big defensins are a family of two-domain AMPs that expanded in mollusks as a result of independent lineage-specific tandem gene duplications, followed by rapid molecular diversification [23,24]. Canonical big defensins harbor an N-terminal hydrophobic domain specific to the peptide family and a C-terminal domain that resembles β-defensins [24,25]. Big defensins have diversified in the oyster *C. gigas* with up to seven distinct sequences described [24]. Among them, *Cg*-BigDef1-3 and *Cg*-BigDef5-6 form two phylogenetically distinct groups [24]. *Cg*-BigDefs are expressed by oyster hemocytes [23]. To date, functional data have only been acquired on *Cg*-BigDef1. This was made possible by developing the chemical synthesis of *Cg*-BigDef1 [26,27]. Synthetic *Cg*-BigDef1 showed a broad range of antibacterial activities against both Gram-positive and Gram-negative bacteria from clinical and environmental collections [27]. A remarkable feature of its mechanism of action was its ability to self-assemble in nanonets and trap and kill bacteria [27].

In this paper, we first tested the in vivo ability of *Cg*-BigDefs to control the commensal oyster microbiota by monitoring microbiota composition in oysters injected with *Cg*-BigDef1. Second, we searched whether primary sequence diversification among oyster big defensins translates into functional diversification. To answer this second question, we cloned the genomic sequences and chemically synthesized *Cg*-BigDef1 and *Cg*-BigDef5, which are representative of sequence diversity. Antimicrobial activity spectra of *Cg*-BigDefs were determined in vitro against a collection of culturable bacteria belonging to the oyster microbiota. Our data support a role for *Cg*-BigDefs in the regulation of oyster microbiota composition and show that the sequence diversity between *Cg*-BigDef1 and *Cg*-BigDef5 generates antimicrobial specificity and synergy against members of the oyster microbiota, including bacteria associated with significant pathologies.

## 2. Results

### 2.1. In Vivo Activity of Cg-BigDef1 on Oyster Commensal Microbiota

**Figure 1:**
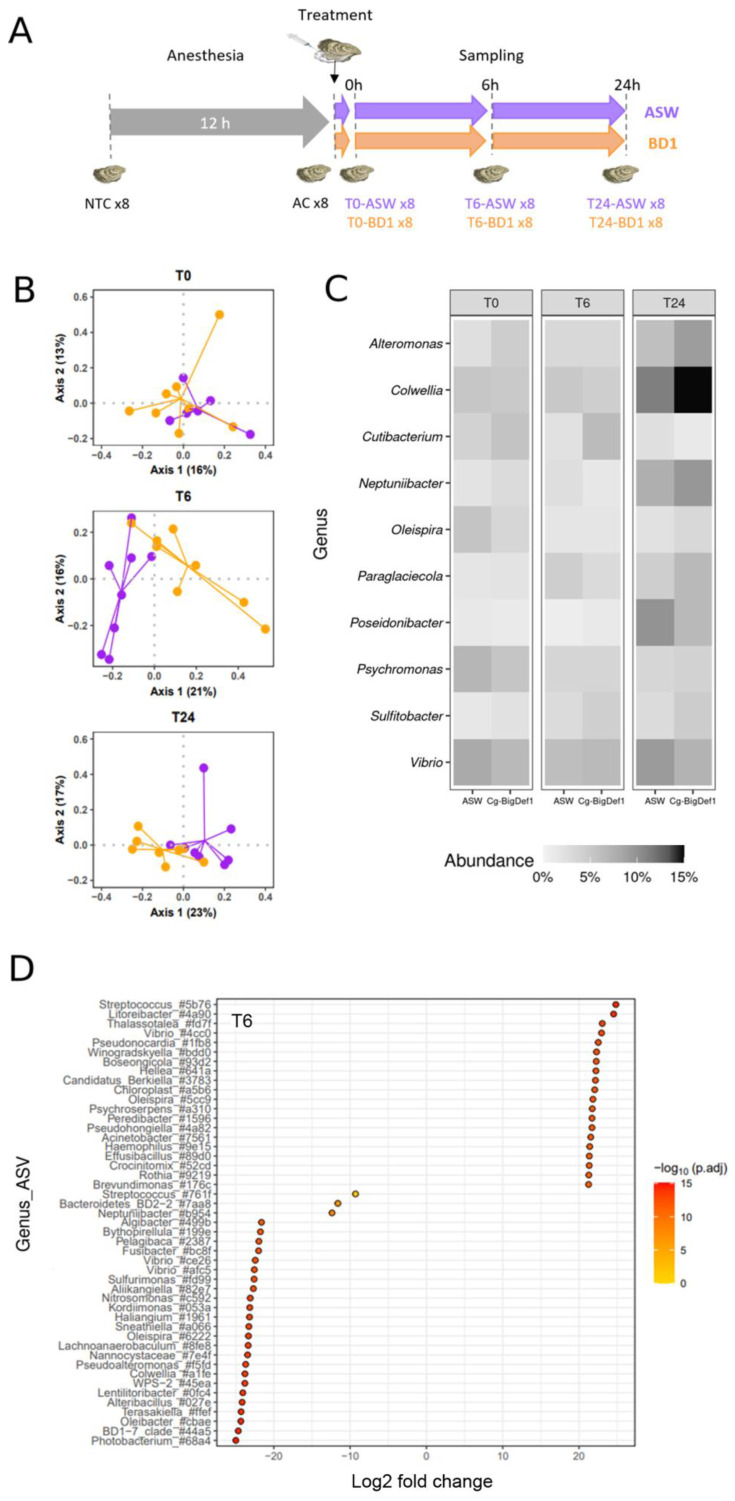
Effects of *Cg*-BigDef1 on β-Diversity and taxonomic composition of oyster microbiota. (**A**): Schematic representation of the experimental design used to test the impact of *Cg*-BigDef1 on the oyster microbiota after injection. From the same batch of oysters, 8 non-treated oysters were collected (NTC), the remaining oysters were anesthetized and 8 oysters were collected after 12 h (AC). The oysters were then injected with either *Cg*-BigDef1 at a final concentration of 5 µM (BD1) or with an equal volume of ASW (control). Oyster sampling was performed 10 min (T0), 6 h (T6) and 24 h (T24) after injection. (**B**): PCoA biplot based on Bray–Curtis distances showing differences between oysters injected with *Cg*-BigDef1 (BD1, orange) or ASW (purple) at T0, T6 and T24. (**C**): Mean relative abundance of bacterial genera in oyster microbiota, grouped according to oyster treatment and time after treatment. The heatmap shows the frequencies of the 10 most abundant bacterial genera in each condition. (**D**): Differential abundance analysis (DESeq2) at the ASV level between oysters injected with ASW and *Cg*-BigDef1 at T6. Each circle represents an ASV showing significant log_2_Foldchange (adjusted *p* value < 0.01) between experimental conditions. Positive log_2_FoldChange means enrichment in *Cg*-BigDef1-injected oysters and negative log_2_FoldChange means enrichment in ASW-injected oysters. Taxa are denoted by their attributed genus followed by the first four characters of the ASV barcode attributed by SAMBA. Note that ASVs without genera annotation were not represented in the figure.

We tested the effect of *Cg*-BigDef1 on oyster commensal microbiota by injecting the synthetic peptide into the adductor muscle (5 µM *Cg*-BigDef1 relative to oyster flesh volume) of anesthetized oysters. An injection of sterile artificial seawater (ASW), i.e., the solvent used for solubilizing synthetic *Cg*-BigDef1, was used as a control (Figure 1A). Since substantial inter-individual variations were observed in oyster microbiota composition [28] and oyster genetics influences microbiota composition [29], we used a pathogen-free oyster family of full siblings for our experiments (i.e., oysters with limited environmental and genetic variation; see Materials and Methods). Microbiota composition was monitored in whole tissue extracts by 16S metabarcoding.

We first verified that anesthesia had no significant effect on the homeostasis of oyster commensal microbiota. To this end, we compared the microbiota of eight non-treated control oysters (NTC, i.e., not anesthetized, not injected with ASW) and eight anesthetized control oysters (AC, i.e., oysters kept dry for 12 h and anesthetized for 2 h). This comparison was performed at time 0 before oysters were injected with *Cg*-BigDef1 or sterile artificial seawater, used as a control. We then examined the effect of *Cg*-BigDef1 on oyster commensal microbiota by comparing the microbiota of eight oysters injected with *Cg*-BigDef1 or ASW (control) at three time points after injection (0, 6, and 24 h) (Figure 1A). To compare microbiota composition over time and conditions, we generated a global dataset from a total of 7,320,778 raw reads obtained by Illumina MiSeq sequencing of the 64 oysters analyzed. Sufficient sequencing depth was confirmed by analyses of rarefaction curves of species richness (Supplementary Figure S1). We retained 6,371,737 sequences corresponding to 632 Amplicon Sequence Variants (ASV) for further analyses after filtering, chimera removal, clustering by dbOTU3, and rare ASVs filtration.

Anesthesia had no significant effect on oyster microbiota. Indeed, AC oysters did not differ from NTC control oysters in terms of α-diversity (measured here by the observed richness and Shannon H indices) nor β-diversity (measured by the Bray–Curtis dissimilarity matrix estimates) (Supplementary Figure S2, Table S1) or relative abundance of the 10 most abundant genera in AC and NTC animals (Supplementary Figure S2, Table S2). Moreover, oysters injected with *Cg*-BigDef1 did not differ from oysters injected with ASW in terms of α-diversity as estimated with the observed richness and Shannon’s H indexes (Supplementary Figure S3).

By contrast, *Cg*-BigDef1 altered the oyster microbiota in terms of β-diversity. This was determined using a final matrix of 632 ASVs distributed among the 48 oyster microbiota samples and the three kinetic points after normalization/rarefaction and removal of low abundance ASVs (less than four reads in at least four individuals). Differences are depicted by principal coordinate analysis (PCoA) based on the Bray–Curtis dissimilarity matrix for T6 and T24 (Figure 1B). Subsequent statistical analyses demonstrated that at T0 (i.e., 10 min after injection with *Cg*-BigDef1 or ASW), oyster microbiota did not differ between conditions (*p* = 0.779). The effect of *Cg*-BigDef1 became visible from T6 (*p* = 0.00064) to T24 (*p* = 0.00488) (PERMANOVA based on 100,000 permutations) (Tables S2 and S3), in agreement with the antimicrobial activity of *Cg*-BigDef1 measurable in vitro within 24 h [27]. No significant differences were observed among the 10 most abundant genera between *Cg*-BigDef1 and ASW-injected oysters (Figure 1C, Supplementary Figure S4). Significant differences were only observed at the ASV level. Overall, at T6, differential abundance analysis identified 156 ASVs, which were significantly enriched or impoverished in *Cg*-BigDef1-treated oysters. Among these, 47 ASVs were affiliated with 44 known genera (Figure 1D). Similar results were obtained at T24 (Supplementary Figure S5). While differences were observed in microbiota composition, the relative abundance of total microbiota did not vary significantly upon *Cg*-BigDef1 treatment, as determined by 16S quantitative PCR (Supplementary Figure S6).

### 2.2. Establishment of a Collection of Culturable Bacteria from C. gigas Microbiota

**Figure 2:**
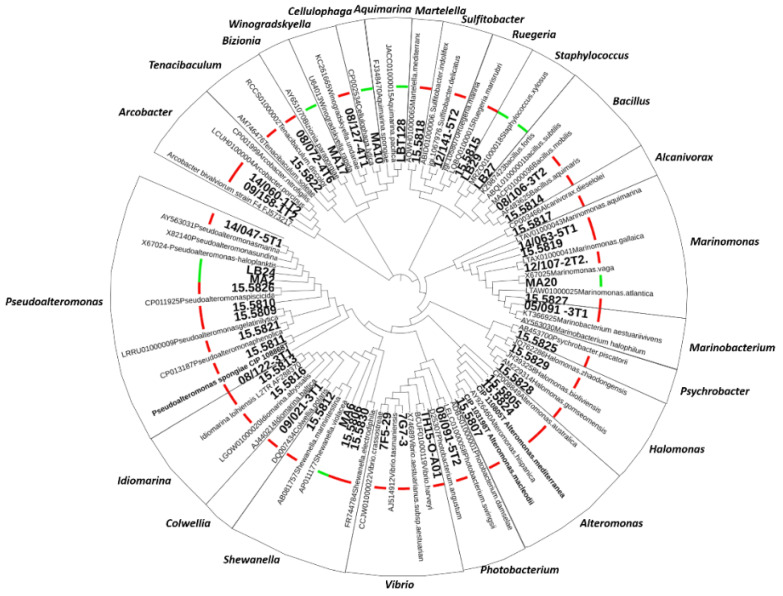
Constitution of a collection of oyster microbiota culturable bacteria. Phylogeny of strains isolated from the *C. gigas* oyster microbiota based on the V3–V4 loop alignment of bacterial 16S rDNA by a Maximum Likelihood method with the Kimura 2-parameter model in MEGA X (295-bp sequences, 105 sequences). Oyster isolates are indicated in boldface. The different genera are indicated. The pathological context at the time of isolation is indicated by a thick bar (red: diseased and green: healthy oysters).

To further investigate the role of *Cg*-BigDefs in controlling the oyster microbiota, we built a collection of culturable bacterial strains representing 21 genera associated with healthy and diseased *C. gigas* [30,31]. Among them, we included genera repeatedly associated with oyster diseases, such as *Arcobacter*, *Aeromonas*, *Marinomonas*, *Marinobacterium*, *Pseudoalteromonas*, *Psychrobacter*, *Sulfitobacter*, *Tenacibaculum*, and *Vibrio* [21,30,32,33]. We obtained 16S rDNA sequences (V3-V4 loop) for 46 bacteria isolated from oysters with known health status (healthy or diseased). In addition, we purchased three type-strains of the genera of interest that were needed as a reference for the MALDI database (one *Pseudoalteromonas* and two *Alteromonas*). All 16S sequences exhibited ≥ 95% identity with a known type-strain sequence included in the analysis (Figure 2). For 41 strains of the 50 strains in the collection, we acquired molecular mass fingerprints by MALDI Biotyping. Strains with taxonomic assignation by 16S phylogeny but no match in MALDI databases were used to enrich the MALDI databases of marine bacteria https://doi.org/10.12770/261d7864-a44c-43ab-b0c6-57fdaf7360ac (accessed on 14 October 2022).

### 2.3. Gene Cloning and Chemical Synthesis of Cg-BigDef1 and Cg-BigDef5

To explore the impact of *Cg*-BigDefs sequence diversification on the control of oyster microbiota, we focused on *Cg*-BigDef1 and *Cg*-BigDef5, which belong to two phylogenetically distinct groups within this peptide family, as shown previously in [24].

**Figure 3:**
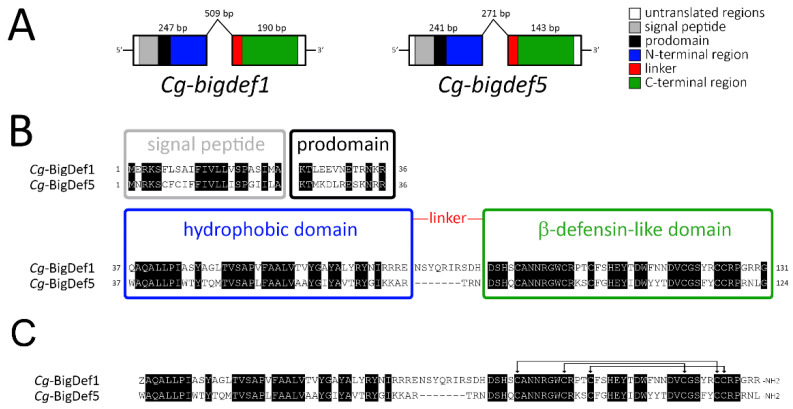
Gene structure and primary sequence of *Cg*-BigDef1 and *Cg*-BigDef5. (**A**): Schematic representation of *Cg*-BigDef1 (GenBank: JN251125) and *Cg*-BigDef5 (GenBank: OP191676) genes. Exons are represented by boxes; introns are represented by lines. The length of each exon/intron is displayed. Grey and black boxes represent nucleotide sequences encoding the signal peptides and prodomains, respectively, while white boxes represent untranslated regions. Blue, red and green boxes represent nucleotide sequences encoding the N-terminal hydrophobic domain, the linker region and the C-terminal β-defensin-like domain of the mature big defensins, respectively. (**B**): Prepropeptides. Alignment of *Cg*-BigDef1 (131 amino acid residues) and *Cg*-BigDef5 (124 amino acid residues) precursor sequences. The signal peptides, prodomains, the hydrophobic (N-terminal) and the β-defensin-like (C-terminal) domains are in boxes. Conserved amino acids are highlighted in black. The linker region is shown in red. (**C**): Putative mature peptides. Alignment of *Cg*-BigDef1 (93 amino acid residues) and *Cg*-BigDef5 (86 amino acid residues). *Cg*-BigDef1 starts with a pyroglutamic acid (Z) and ends with an amidated arginine (R-NH2). *Cg*-BigDef5 ends with an amidated leucine (L-NH2). Both *Cg*-BigDef1(1–93) and *Cg*-BigDef5(1–86) are folded by three intramolecular disulfide bridges as shown by arrows.

We first cloned the gene encoding *Cg*-BigDef5 (*Cg-bigdef5* gene; GenBank: OP191676). Two distinct exons were found to encode the two putative domains of the molecule (Figure 3A), as previously found in *Cg-bigdef1* [27]. The first exon of *Cg-bigdef5* encodes the predicted signal peptide (or predomain, 23 residues), the prodomain (13 residues), and the N-terminal domain of the mature *Cg*-BigDef5 (42 residues). The second exon encodes a short linker (3 residues) and the C-terminal β-defensin-like domain (42 residues), with the canonical spacing of cysteines for big defensins [Cys-Xaa_(4–14)_-Cys-Xaa_(3)_-Cys-Xaa_(13–14)_-Cys-Xaa_(4–7)_-Cys-Cys] [27] (Figure 3B). After posttranslational modifications (which include removal of the preprodomain, oxidation of the three disulfide bridges, glutamine to pyroglutamic acid conversion, and C-terminal amidation by removal of a glycine residue), the calculated molecular weight (MW) of *Cg*-BigDef1 was 10,692 Da (93 amino acids). The calculated MW for *Cg*-BigDef5 was 9977 Da (86 amino acids) after the removal of the preprodomain, disulfide bridge oxidation, and C-terminal amidation by removal of a glycine residue (Figure 3C). Overall the two mature peptides show 62.8 % identity (54/86 identical residues) with a calculated positive net charge of +6 and +7 at pH = 7.4 for *Cg*-BigDef1 and *Cg*-BigDef5, respectively.

The N-terminal domain of both peptides is hydrophobic and positively charged in the region preceding the linker due to repeats of basic residues such as arginine in *Cg*-BigDef1 and lysine in *Cg*-BigDef5. One remarkable difference between the two big defensins is the length of the linker that connects the two domains, with 10 amino acid residues in *Cg*-BigDef1 and only three amino acid residues in *Cg*-BigDef5.

*Cg*-BigDef1 and *Cg*-BigDef5 were synthesized using a combination of solid-phase peptide synthesis, native chemical ligation, and oxidative folding as previously described for *Cg*-BigDef1 [26,27] (see Supplementary Figure S6 for HPLC and mass spectrometry characterization). Synthetic *Cg*-BigDef1 (1–93) corresponds to mature *Cg*-BigDef1 (Figure 3C) [27]. Synthetic *Cg*-BigDef5 (1–86) corresponds to mature *Cg*-BigDef5 (Figure 3C) with a substitution of Met14 by norleucine (Nle) (this study). Detailed optimization of *Cg*-BigDef5 (1–86) synthesis and NMR structure determination will be described elsewhere [34].

### 2.4. Specificity, Synergy, and Complementary Broad-Spectrum Activity of Cg-BigDef1 and Cg-BigDef5 against Bacteria from the Oyster Microbiota

We used synthetic *Cg*-BigDef1 and *Cg*-BigDef5 to study their antibacterial activities against bacteria from the microbiota of *C. gigas*, including strains relevant to oyster infections. All assays were performed under physiological conditions, i.e., at a high salt concentration (400 mM NaCl).

*Cg*-BigDef1 showed antibacterial activity against 11/26 strains from *C. gigas* microbiota (Table 1). In total, *Cg*-BigDef1 was bactericidal against six strains. High bactericidal activity was observed against *Bacillus* sp. 15.5814 (MIC = 40 nM, MBC = 310 nM). In addition, bactericidal activity was recorded against *Alcanivorax* sp. 15.5817, *Alteromonas* sp. 15.5805, *Halomonas* sp. 15.5829, *Pseudoalteromonas* sp. 15.5809 and *Winogradskyella* sp. 08.27-4T1 with MICs in the range of 1.25–10 µM and MBCs in the range of 5–10 µM. Up to 10 µM, *Cg*-BigDef1 was inhibitory but not bactericidal against five additional strains, namely *Aquimarina* sp. LTB 128, *Marinomonas* sp. 15.5827, *Martellela* sp. 15.5818, *Shewanella* sp. 15.5830, and *Tenacibaculum* sp. 08.072-4T6 with MICs ranging from 1.25 to 5 µM (Table 1). All strains except *Aquimarina* sp. LTB 128 were isolated from diseased oysters.

*Cg*-BigDef5 tended to be less active than *Cg*-BigDef1. Still, it showed antibacterial activity against 9/26 tested strains from the *C. gigas* oyster microbiota (Table 1). The highest activity was recorded against *Marinomonas* sp. 14.063 with a MIC of 0.6 µM. *Cg*-BigDef5 was also active against *Alteromonas* sp. 15.5805, *Aquimarina* sp. LTB 128, *Bacillus* sp. 15.5814, *Marinobacterium* sp. *05.091-3T1*, *Martellela* sp. 15.5818, *Ruegeria* sp. 15.5815, *Shewanella* sp. 15.5830, and *Sulfitobacter* sp. 12.141-5T2 with MICs ranging from 1.25 to 10 µM. No bactericidal activity was observed at concentrations ≤10 µM.

Overall, *Cg*-BigDef1 and *Cg*-BigDef5 were both active at a salt concentration (400 mM NaCl) pertinent to marine bacteria. They showed strain specificity and complementary activity spectra against the marine strains of the oyster microbiota collection: five strains were susceptible to both peptides, whereas six and four strains were only susceptible to *Cg*-BigDef1 and *Cg*-BigDef5, respectively. Only *Cg*-BigDef1 was active against *Marinomonas* sp. 15.5827, *Pseudoalteromonas* sp. 15.5809, and *Tenacibaculum* sp. 08.072-4T6, *Alcanivorax* sp. 15.5817, *Halomonas* sp. 15.5829, *Winogradskyella* sp. 08.27-4T1. Conversely, only *Cg*-BigDef5 was active against *Marinobacterium* sp. 05.091-3T1 and *Marinomonas* sp. 14.063, *Ruegeria* sp. 15.5815 and *Sulfitobacter* sp. 12.141-5T2. Both *Cg*-BigDef1 and *Cg*-BigDef5 were active against *Alteromonas* sp. 15.5805. These data show that *Cg*-BigDef sequence diversity extends the activity spectrum at the inter-genera level. The example of *Marinomonas* sp. strains, which are susceptible to different *Cg*-BigDefs, highlights an undiscovered specificity of *Cg*-BigDefs and illustrates that their sequence diversity extends their activity spectrum at an intra-genus level as well. It is important to note that all the strains mentioned here have a significant role in oyster health: they have been isolated from OsHV-1-infected oysters https://doi.org/10.12770/0d529567-92fd-4dcd-9d9c-70e98ab6f772 (accessed on 14 October 2022), and several of them belong to a set of conserved genera that proliferate during OsHV-1-induced dysbiosis [30].

Finally, we tested the synergies of *Cg*-BigDef1 and *Cg*-BigDef5, by the checkerboard microtiter assay, against a Gram-positive and a Gram-negative strain displaying the lowest MICs for both peptides. The two big defensins acted synergistically against both strains. Indeed, synergy was recorded against the Gram-negative *Alteromonas* sp. 15.5805, with a fractional inhibitory concentration (FIC) index value of 1 (Table 1). Strong synergy was observed against the Gram-positive *Bacillus* sp. 15.5814 with an FIC value of 0.35 (Table 1).

To summarize, *Cg*-BigDef1 and *Cg*-BigDef5 exhibit a broad activity spectrum. They show strain specificity, as well as complementary activities. Together they inhibit the growth of 15/26 strains tested. Finally, they act synergistically against both Gram-positive and Gram-negative bacteria. Altogether, these data show that sequence diversification of *Cg*-BigDefs has generated antimicrobial specificity and extended the activity spectrum of the peptide family against marine bacteria from the oyster microbiota, including strains associated with oyster pathologies.

## 3. Discussion

We found that oyster big defensins (*Cg*-BigDefs), a family of AMPs that have widely diversified in mollusks, can alter oyster microbiota composition in vivo as a result of direct antimicrobial activity against members of the oyster microbiota. Remarkably, we observed that sequence diversification had generated antimicrobial specificity as well as synergy between *Cg*-BigDef1 and *Cg*-BigDef5, thereby extending the activity spectrum of the peptide family and increasing its potency.

Until now, it was largely unknown whether AMPs could shape the microbiota of mollusks, while this had been demonstrated in other animal phyla, particularly mammals [4], cnidarians [14], and insects [6]. Furthermore, when available, antimicrobial data have been largely acquired on microorganisms unrelated to molluscan health [27,36,37]. The lack of knowledge on immune/microbiota interactions in mollusks is due to several methodological obstacles and knowledge gaps. Among them, it is worth mentioning (i) the only recent description of molluscan microbiomes, accelerated by facilitated access to next-generation sequencing (for oysters, see [18,19,31]); (ii) the lack of well-characterized culturable microbiota; and (iii) difficulties in producing a sufficient amount of high-quality AMPs and in developing efficient and reliable tools for gene knock-in, knock-out, and knock-down in several molluscan species. These difficulties were circumvented in this work by the chemical synthesis of pure big defensins from *C. gigas* according to our previously described procedure [26] and the construction of a collection of culturable bacteria isolated from oysters with known health status (identification by 16S phylogeny and MALDI Biotyping). With such tools, we showed that *Cg*-BigDefs have broad-spectrum activities against bacterial strains from the oyster microbiota, including strains associated with major infectious diseases in oysters. In line with these observations, in vivo, *Cg*-BigDef1 induced significant changes in oyster microbiota β-diversity. This is consistent with in vivo results recently obtained by Schmitt and collaborators in the scallop *Argopenten purpuratus* [22]. The authors showed that the big defensin *Ap*BD1 and the bactericidal/permeability-increasing protein *Ap*LBP/BPI1 have the potential to shape the hemolymph microbiota of the scallop, particularly by regulating the proliferation of γ-proteobacteria. Our present work shows that changes in microbiota composition observed in vivo are linked to direct antimicrobial activities of *Cg*-BigDefs against bacteria belonging to the microbiota. Similar to human α-defensin HD-5 in the mice gut [38], *Cg*-BigDef1 did not alter the overall bacterial load in oysters. Moreover, *Cg*-BigDef1 had no negative effects on oyster microbiota diversity, probably due to the specificity of the peptides, which as host-defense effectors, have evolved to acquire antimicrobial activity against given bacterial strains without disrupting the entire oyster microbiota. Supporting this hypothesis, microbiota alterations were mainly visible at the ASV taxonomic level, indicating high specificity. For instance, upon treatment with *Cg*-BigDef1, we observed a reduced amount of *Vibrio* (γ-proteobacteria), which was in agreement with the in vitro activity of *ApBD1* in the scallop [22]. Changes were visible in whole-tissue microbiota. However, microbiota composition was shown to vary significantly between oyster tissues (hemolymph, gut, gills, mantle) [28]. Therefore, it is likely that more contrasting effects of *Cg*-BigDefs occur on specific tissue microbiota, particularly in the hemolymph, which carries the *Cg*-BigDef-producing cells, the hemocytes [23]. With mounting evidence on the regulatory role of AMPs on host-microbiota across animal phyla, including mollusks ([22], this study), one key question to be addressed in the future is how this affects the functions the microbiota serve in their host tissues.

A striking feature of the evolutionary history of big defensins is their extensive diversification in some molluscan species, particularly the oyster *C. gigas* and the mussels *Mytilus galloprovinciallis* and *Dreissena rostriformis*, while they did not diversify in other species (e.g., the scallop *A. purpuratus*) [24]. The functional consequences of this diversification have remained unexplored. Our present results demonstrate that sequence diversification has generated specificity and synergy among *Cg*-BigDefs, as evidenced by two members of the *Cg*-BigDef family, *Cg*-BigDef1 and *Cg*-BigDef5. Antibacterial specificity was observed from the bacterial genus down to the strain level within a given genus. Depending on bacterial strains, *Cg*-BigDefs were bactericidal or simply inhibitory, with contrasting MICs, from 40 nM to 10 µM. This suggests that distinct mechanisms of action can underpin *Cg*-BigDefs activities against the diversity of bacteria encountered in the oyster microbiota. Activities in the nanomolar range are consistent with receptor-mediator activities [36,39], while activities in the micromolar range are typically reported for membrane-active AMPs [40]. In oyster defensins (*Cg*-Defs), which have also diversified in oysters, we previously observed that sequence variation altered the activity of the peptide (more or less potent) without affecting the peptide range of activity [35]. Here, we also showed that sequence diversification was key to generating antimicrobial synergy between two members of the *Cg*-BigDef family. Similarly, sequence diversification generated synergy in two other families of oyster AMPs, the defensins *Cg*-Defs and the proline-rich peptides *Cg*-Prps [35]. Although not studied in the present article, synergy also occurred between AMP families, as observed between the bactericidal permeability-increasing protein *Cg*-BPI, *Cg*-Prps, and *Cg*-Defs in the oyster *C. gigas* and between Attacins and Diptericins in the insect *D. melanogaster* [41]. Thus, the in vivo effects of *Cg*-BigDefs on the shaping of oyster microbiota are likely to extend well beyond the observations in this paper, where we tested the effects of only one member of the *Cg*-BigDef family. This was also the case in the *A. purpuratus* scallop study. However, in scallops, unlike other molluscan species (oysters and mussels) big defensins have not diversified and the activity of *Ap*BD1 recapitulates that of the entire AMP family. Overall, we have highlighted an important role for sequence diversification in increasing the antimicrobial potential of oyster *Cg*-BigDefs, by generating both antimicrobial specificity and synergy, an observation that extends at least to two additional peptide families, *Cg*-Defs and *Cg*-Prps. We can hypothesize that some species of bivalve mollusks, such as oysters, have diversified their repertoire of AMPs to increase their adaptive potential while constantly exposed to diversified microbial communities.

**Figure 4:**
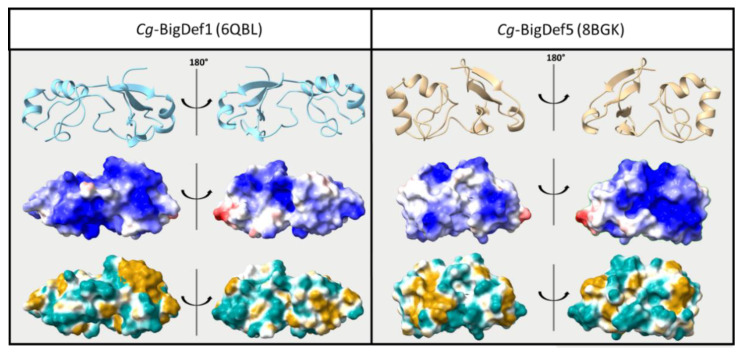
Structure comparison of *Cg*-BigDef1 and *Cg*-BigDef5. Top—Cartoon representation. Middle—Electrostatic potential on the accessible surface of the proteins with red representing negative charges and blue representing positive charges. Bottom—Hydrophobicity potential on the accessible surface of proteins with light blue representing hydrophilic properties and brown representing hydrophobic properties. Both electrostatic and hydrophobic potential were determined using ChimeraX software [42,43].

While sequence diversification was shown to be a major asset in terms of antimicrobial defenses, we still do not know how antimicrobial specificity is generated. We have shown that changes in primary structure between *Cg*-BigDef1 and *Cg*-BigDef5 (62.8% sequence identity) produced antibacterial specificity. *Cg*-BigDef1 and *Cg*-BigDef5 have similar biophysical parameters in terms of size (86–93 amino acids) and positive net charge at neutral pH (+6 to +7 at pH = 7.4, i.e., oyster physiological pH), with conserved domains, as recently determined by NMR ([34]; Figure 4 top panel). The position and pairing of cysteines are also similar. A major difference observed between *Cg*-BigDef1 and *Cg*-BigDef5 was the length and primary sequence of the linker region connecting the N-terminal hydrophobic domain and the C-terminal β-defensin-like domain, whereas the five residues linker of *Cg*-BigDef5 is exposed to the solvent, the ten residues linker of *Cg*-BigDef1 plays a key role in the 3D compaction of the protein by being buried at the interface of the two domains and locking their relative orientation [27]. In *Cg*-BigDef1 and *Cg*-BigDef5, the orientation of the N- and C-terminal domains differs by around 100° (dihedral angle value between the β-sheet of the N-term domain and the last strand of the β-sheet of the C-term domain (Figure 4 top panel), leading the α-helix of the C-term domain not involved in the interaction interface between the two domains. We also looked at surface properties, as the surface charge is considered critical for the interactions of AMPs with bacteria [42]. However, no real quantitative difference can be observed between *Cg*-BigDef1 and *Cg*-BigDef5. Both are highly cationic, and positive charge repartition is shared by the N-terminal and C-terminal domains, yet the positive surface of each molecule is on opposite sides (Figure 4 middle panel). Since the salt concentration in the oyster is very high (similar to seawater), charges may be shielded and might not be the primary type of interaction that is important for bacterial interaction. Instead, the hydrophobicity of the big defensin surface could play a major role in how the molecules approach their target. As seen in Figure 4 (bottom), *Cg*-BigDef1 displays a more hydrophobic C-terminal domain than *Cg*-BigDef5, whereas *Cg*-BigDef5 displays a more hydrophobic N-terminal domain than *Cg*-BigDef1.

We are still unable to explain which structural determinants play a role in the specificity of *Cg*-BigDefs. For instance, we do not know whether residues exposed in the linker (i.e., the most diversified residues) play a role in the interaction with microbes. Another unresolved issue is the salt stability of *Cg*-BigDef activity at high salt concentrations. Indeed, both *Cg*-BigDef1 and *Cg*-BigDef5 were active against a wide range of marine bacteria at 400 mM NaCl, in agreement with previous findings for *Cg*-BigDef1 [27]. This stability of the antimicrobial activity is unique and essential for the peptides to participate as direct effectors in oyster antimicrobial defense. While many questions are still open on the structure-activity relationships of big-defensins and their domains, the molecular tools are now available to unveil the consequences of sequence variation in the interactions of *Cg*-BigDefs domains with the bacteria and/or in nanonet assembly. The same applies to bacteria from the oyster microbiota, with a collection of culturable bacteria that will be highly useful for testing functional hypotheses.

### Conclusions

We have demonstrated that sequence diversification in *Cg*-BigDefs has helped to improve oyster defense against pathogens and to control oyster-associated bacterial communities. Indeed, we highlighted an undiscovered specificity and synergy between *Cg*-BigDefs, which broadened their activity spectrum. These results pave the way for future studies on the mechanism of action of big defensins, which may vary depending on bacterial targets.

## 4. Materials and Methods

### 4.1. Chemicals and Reagents

MilliQ water (Merck Millipore, Billerica, MA, USA) was used. LC-MS-grade acetonitrile (ACN) was obtained from Carlo-Erba Reagents (Val de Reuil, France). LC-MS-grade formic acid (FA), trifluoroacetic acid (TFA), and alpha-cyano-4-hydroxycinnamic acid (4-HCCA) were purchased from Sigma-Aldrich (St. Louis, MO, USA).

### 4.2. Oysters

Oysters with limited genetic diversity were obtained as follows. Genitor oysters were collected in 2015 from the Le Dellec area in Brest bay, which is devoid of shellfish farming. The first generation of full siblings was produced, named F14, as described in [21]. From this family, two oysters were used to generate a second generation of full siblings, referred to as F14V (Decicomp project ANR-19-CE20-004). Offspring were kept at the Ifremer hatchery in Argenton (France) up to day 40. Then, they were grown at the Ifremer station in Bouin (France) until they were 10 months old.

### 4.3. Bacterial Strains and Culture Conditions

Strains and media are listed in the Ifremer Sextant catalog at https://doi.org/10.12770/0d529567-92fd-4dcd-9d9c-70e98ab6f772 (accessed on 14 October 2022).

Isolation of bacteria from oyster flesh and antibacterial assays were performed in Zobell medium at 20 °C. Zobell medium is composed of artificial seawater (ASW) [44] supplemented with 0.4% bactopeptone and 10% yeast extract, pH 7.8.

Bacteria were isolated from the flesh of live oysters affected by the Pacific Oyster Mortality Syndrome (susceptible families F11, F14, and F15 from the Decipher project ANR-14-CE19-0023) [21]. Additional bacteria isolated from diseased oysters were provided by the French National Reference Laboratory (Ifremer, La Tremblade, France). Finally, bacteria isolated from healthy commercial or wild oysters were included.

### 4.4. Molecular Phylogeny Based on 16S RNA

Taxonomic assignment down to the genus was performed for each strain by molecular phylogeny based on the sequence of the V3-V4 region of 16S rRNA obtained by Sanger sequencing. In order to consolidate the phylogenetic tree, at least one Genbank reference sequence corresponding to a Type (T) strain per genus of interest was added from the NCBI database (Table S4). The 95 sequences were trimmed at 405 bp (V3-V4 loop) using BioEdit and aligned using ClustalW. The phylogenetic tree was constructed by the Maximum Likelihood method with the Kimura 2-parameter model [45] using MEGA X software [46] and annotated using ITOL software [47]. The branches are supported by the bootstrap method with 500 iterations.

### 4.5. Identification of Bacterial Isolates by Matrix-Assisted Laser Desorption Ionization Mass Spectrometry (MALDI Biotyping)

MALDI Biotyping was used to confirm 16S taxonomic assignments or, when the libraries required it, to enrich them with new bacteria absent from MALDI libraries (common for marine strains). For these purposes, a protocol coupling inactivation with 75% ethanol and extraction with 70% formic acid was performed based on the MALDI Biotyper^®^ protocol (Bruker Daltonics, Bremen, Germany). Briefly, from each plate, one isolated colony was suspended in MilliQ water in 1.5 mL Eppendorf tubes. Ethanol (100%) was added to the suspension, and the tubes were centrifuged twice (13,000 rpm, 2 min). Subsequently, 10 μL of a 70% formic acid solution was added to the pellet. In order to complete the extraction, 10 μL of pure acetonitrile was added. One microlitre of each extract was deposited three times (technical replicates) on a MALDI target (Bruker Daltonics, Bremen, Germany), air-dried, and coated with 1 μL of fresh alpha-cyano-4-hydroxycinnamic acid matrix in a saturating amount in a solution of 50% ACN and 2.5% TFA (Bruker Daltonics, Bremen, Germany). The MALDI MS spectra of these spots were acquired with an Autoflex III Smartbeam MALDI-TOF MS, recording masses ranging from 2000 to 20,000 Da using standard parameters (flexControl 3.4, Bruker Daltonic, Bremen, Germany), and interrogated against the existing databases. For bacteria species not present in the existing MALDI Biotyper^®^ reference mass spectra libraries, a reference spectrum was created and entered in our local database, as follows: the bacterial extract was spotted 8 times, each spot analyzed three times, for a total of twenty-four recorded spectra per bacterial strain. After manual checking, the twenty better spectra were transformed into an average spectrum by the MBT Compass Explorer software. The BTS (bacterial test standard) serves as a calibrator and contains *Escherichia coli* extract. The reference libraries used for the analysis are the official Bruker MALDI Biotyper^®^ spectral library (MBT reference library https://www.bruker.com/en/products-and-solutions/microbiology-and-diagnostics/microbial-identification/maldi-biotyper-library-ruo.html; accessed on 10 October 2022) and the freely available EnviBase exclusively dedicated to the identification of potentially pathogenic *Vibrio* in marine mollusks (seanoe.org, accessed on 10 October 2022) [48].

### 4.6. Molecular Cloning and Sequence Data Analysis

The *Cg*-BigDef5 gene was PCR-amplified using specific primers (Fw: 5′-AATCAAGTCAACATGAACAG-3′; Rv: 5′-TTATCCTAGATTTCTAGGTC-3′) based on a transcript sequence previously found in publicly available databases [24], cloned into a pGEM-T Easy vector (Promega) and then sequenced using the Sanger dideoxy methodology (Applied Biosystems 3500 Series Genetic Analyzer). Exon–intron boundaries were defined by the alignment of cDNA and genomic sequences. Nucleotide sequences were manually inspected and translated using the ExPASy Translate Tool http://web.expasy.org/translate/ (accessed on 1 September 2022). Prediction of signal peptides and other posttranslational processing was carried out using the ProP 1.0 server https://services.healthtech.dtu.dk/service.php?ProP-1.0 (accessed on 1 September 2022), while the theoretical isoelectric point (p*I*) and molecular weight (MW) of the mature peptides were calculated using the Expasy ProtParam Tool http://web.expasy.org/protparam/ (accessed on 1 September 2022). Multiple alignments of amino acid sequences were generated using MUSCLE with default parameters https://www.ebi.ac.uk/Tools/msa/muscle/ (accessed on 1 September 2022).

### 4.7. Peptide Synthesis and Net Charge Calculation

*Cg*-BigDef1 was synthesized as already described, using a combination of solid-phase chemical synthesis and native chemical ligation (NCL) followed by a thermodynamically controlled oxidative folding step [26,27,49]. *Cg*-BigDef5 was obtained following a similar synthetic scheme (see Supplemental Figure S5 and [34] for optimization and details, as well as for 3D structure determination by NMR).

Peptide net charges at pH = 7.4 (oyster physiological pH) were predicted using the IPCprotein pKa dataset [50]. *Cg*-BigDef1 net charge = +6.05 (6.00 negative charges and 12.05 positive charges, calculated taking into account 12 Arg, 4 Asp, 2 Glu, 3 His, and 8 Tyr). *Cg*-BigDef5 net charge = +6.95 (4.00 negative charges and 10.95 positive charges, calculated taking into account 7 Arg, 3 Asp, 1 Glu, 2 His, 3 Lys, 8 Tyr, and the N-terminal amine group).

### 4.8. Determination of the Minimum Inhibitory Concentrations (MIC), Minimum Bactericidal Concentrations (MBC), and Synergy

*Cg*-BigDef1(1-93) and *Cg*-BigDef5(1-86) were dissolved in MilliQ water at a concentration of 200 µM. Peptide concentration control was performed with a NanoDrop One spectrometer (Thermo Fisher Scientific, Waltham, MA, USA) using a molar epsilon at 280 nm of 23,295 M^−1^.cm^−1^ for *Cg*-BigDef1 (peptide batch ARO-I-78) and of 34,295 M^−1^.cm^−1^ *Cg*-BigDef5 (peptide batch ARO-I-113).

MIC and MBC values were determined as previously described [35]. Briefly, big defensins stock solutions were serially diluted in sterile MilliQ water. A total of 10 µL of peptides were incubated with 90 µL of bacterial suspension, brought to the exponential growth phase, and adjusted to A_600_ = 0.001 in Zobell medium at 20 °C. Bacteria were grown under shaking in a sterile, non-pyrogenic polystyrene 96-well plate (Falcon). Growth was monitored at 600 nm on a TECAN spectrophotometer with one measurement/h over 24 h. MIC values are expressed as the lowest concentration tested (µM) that results in 100% growth inhibition. For the determination of MBCs, after a 24 h incubation, 100 µL of each well were plated on Zobell agar medium at 20 °C. MBC values are expressed as the lowest concentration tested (µM) for which no colonies could be counted on a Petri dish.

Synergies between *Cg*-BigDef1 and *Cg*-BigDef5 were measured as previously described using the checkerboard microtiter assay, which enables highlighting a potential reduction of the MIC values of each peptide when used in combination. In this assay, 2-fold serial dilutions of one peptide are tested against 2-fold serial dilutions of the other peptide. Results are expressed by calculating fractional inhibitory concentration (FIC) index values [35].

### 4.9. Microbiota Modifications Induced by Cg-BigDef1 In Vivo

A biparental family of juvenile *C. gigas* oysters (family F14-V, 10 months old, average wet weight of flesh 200+/−27 mg) was used in in vivo assays. All oysters were maintained under controlled biosecurity conditions to ensure their specific pathogen-free status. For anesthesia, oysters were kept for 12 h outside seawater tanks and anesthetized two hours before the experiment in seawater containing 50 g/L MgCl_2_ [51]. Control animals (n = 8) were collected before (NTC, non-treated controls) and after the entire anesthesia procedure (AC, anesthesia controls). Before injection into oysters, *Cg*-BigDef1 was dissolved in sterile ASW at a concentration of 20 µM. Concentration was verified as described above for MIC and MBC determination. Injection of *Cg*-BigDef1 (50 µL) was performed right after anesthesia by injection into the oyster adductor muscle to reach a final concentration of 5 µM of *Cg*-BigDef1 in oyster flesh. An injection of 50 µL sterile ASW was used as a control treatment. Oysters (n = 8 per condition) were sampled 10 min (T0), 6 h (T6), and 24 h (T24) after injection. For oyster sampling, shells were removed, and flesh was recovered and snap-frozen in liquid nitrogen. Individual oysters were ground in liquid nitrogen in 50 mL stainless steel bowls with 20-mm-diameter grinding balls (Retsch MM400 mill) and stored at −80 °C until DNA extraction. DNA extraction was performed as described in [21] using the Nucleospin tissue kit (Macherey-Nagel, Düren, Germany). DNA concentration and purity were checked with a NanoDrop One (Thermo Fisher Scientific, Waltham, MA, USA).

### 4.10. 16S rRNA Metabarcoding

Bacterial metabarcoding was performed using 16S rRNA gene amplicon sequencing. Libraries were generated using the Illumina two-step PCR protocol targeting the V3-V4 region (341F: 5′-CCTACGGGNGGCWGCAG-3′; 805R: 5′-GACTACHVGGGTATCTAATCC-3′) [52]. Sequencing was performed at the Bioenvironment Platform (University of Perpignan). A total of 64 libraries were paired-end sequenced with a 2 × 250 bp read length on a MiSeq system (Illumina) according to the manufacturer’s protocol. Raw sequence data are available in the SRA database BioProject ID https://doi.org/10.12770/c960676e-2515-46f0-a313-4a91ac91908a (accessed on 17 October 2022).

Sequencing data were processed using the SAMBA pipeline v3.0.1. The SAMBA workflow, developed by the SeBiMER (Ifremer’s Bioinformatics Core Facility), is an open-source modular workflow to process eDNA metabarcoding data. SAMBA is developed using the NextFlow workflow manager [53]. All bioinformatics processes are mainly based on the use of the next-generation microbiome bioinformatics platform QIIME 2 [54] (version 2020.2) and the approach of grouping sequences in ASV (Amplicon Sequence Variants) using DADA2 v1.14, [55]). Taxonomic assignment of ASVs was performed using a Bayesian classifier trained with the Silva database v.138 using the QIIME feature classifier [56]. Statistical analyses were also performed with R (R Core Team, 2020) using the R packages Phyloseq v1.38.0 [57] and Vegan v2.6-2 [53].

For α-diversity, we used the full data set to analyze differences in regularity (calculated as H/ln (S), where H is the Shannon–Wiener index, and S is species richness) and species richness (total number of species) using SAMBA pipeline and ANOVA.

For β-diversity, the ASV matrix of all 64 libraries was preliminarily normalized. Briefly, after verification of the rarefaction curves produced with the ggrare function [57]), libraries were sub-sampled to 45,361 reads using the rarefy_even_depth function. The normalized ASV matrix was then filtered for low-abundance ASVs to limit the prevalence of putative artifacts due to sequencing errors. For this purpose, only ASVs with at least four reads in at least four samples were retained. We then retained samples associated with ASW and *Cg*-BigDef1 experimental conditions at T0, T6, and T24. The variation in microbiota composition was then investigated using principal coordinate analyses (PCoA) based on Bray–Curtis distances at each kinetic point. Putative differences between groups were assessed by statistical analyses (Permutational Multivariate Analysis of Variance-PERMANOVA) using the adonis2 function implemented in vegan [58].

The mean relative abundance of the 10 most abundant bacterial genera in the oyster microbiota was also estimated. Results were graphically represented by a heatmap. We used the STAMPS software [59] to represent an extended error bar. Statistical differences were assessed by Welch’s t-test with the Benjamini–Hochberg procedure, which controls the false discovery rate (FDR).

Finally, we used DESeq2 v1.36.0 [60] to identify ASVs whose abundance significantly varies in oysters injected with *Cg*-BigDef1 or ASW (control) for the last kinetic point (i.e., T24). Differential abundance was analyzed using a negative binomial method implemented in the DESeq2 package as recommended by [57]. For this latter analysis, we only considered ASVs with an adjusted *p* value < 0.01. Note that ASVs lacking genera annotation and qualified as “unknown” were not considered for result interpretation.

### 4.11. Quantification of Total 16S Bacterial DNA

Total of 16S bacterial DNA was quantified by quantitative PCR (qPCR). All amplification reactions were analyzed using a Roche LightCycler 480 Real-Time thermocycler (qPHD-Montpellier GenomiX platform, Montpellier University, Montpellier, France). The total qPCR reaction volume was 1.5 μL and consisted of 0.5 μL DNA (30 ng.μL^−1^) and 0.75 μL LightCycler 480 SYBR Green I Master mix (Roche) containing 0.5 μM PCR primer (Eurogenetec SA). Primers used for total bacteria were 341F 5′-CCTACGGGNGGCWGCAG-3′ and 805R 5′-GACTACHV GGGTATCTAATCC-3′, which target the 16S variable V3V4 loops [52]. A Labcyte Acoustic Automated Liquid Handling Platform (ECHO) was used for pipetting into the 384-well plate (Roche). A LightCycler^®^ 480 Instrument (Roche) was used for qPCR with the following program: enzyme activation at 95 °C for 10 min, followed by 40 cycles of denaturation (95 °C, 10 s), hybridization (60 °C, 20 s) and elongation (72 °C, 25 s). A melting temperature curve of the amplicon was then performed to verify the specificity of the amplification. Relative quantification of 16S bacterial DNA copies was calculated by the 2^−ΔΔCq^ method [61] using the mean of the measured cycle threshold values of a reference gene (*Cg*-EF1α (elongation factor 1α), GenBank: AB122066), as a calibrator.

## Figures and Tables

**Table 1:** Activity Spectrum and Synergy of *Cg*-BigDef1 and *Cg*-BigDef5 against bacteria associated with disease in the oyster.

Strain	*Cg*-BigDef1 (1–93)		*Cg*-BigDef5 (1–86)		FIC Index
	MIC (µM)	MBC (µM)	MIC (µM)	MBC (µM)	
*Alcanivorax* sp. 15.5817	**2.50**	**5**	>10	>10	*nt*
*Alteromonas* sp. 15.5805	**1.25**	**5**	**2.50**	>10	**1**
*Aquimarina* sp. LTB 128	**5**	>10	**2.50**	>10	*nt*
*Bacillus* sp. 15.5814	**0.04**	**0.31**	**1.25**	>10	**0.35**
*Halomonas* sp. 15.5829	**2.50**	**10**	>10	>10	*nt*
*Marinobacterium* sp. 05.091-3T1	>10	>10	**2.50**	>10	*nt*
*Marinomonas* sp. 14.063	>10	>10	**0.60**	>10	*nt*
*Marinomonas* sp. 15.5827	**2.50**	>10	>10	>10	*nt*
*Martellela* sp. 15.5818	**2.50**	>10	**1.25**	>10	*nt*
*Pseudoalteromonas* sp. 15.5809	**10**	**10**	>10	>10	*nt*
*Ruegeria* sp. 15.5815	>10	>10	**10**	>10	*nt*
*Shewanella* sp. 15.5830	**1.25**	>10	**10**	>10	*nt*
*Sulfitobacter* sp. 12.141-5T2	>10	>10	**2.50**	>10	*nt*
*Tenacibaculum* sp. 08.072-4T6	**1.25**	>10	>10	>10	*nt*
*Winogradskyella* sp. 08.27-4T1	**1.25**	**5**	>10	>10	*nt*

MIC values reported in micromoles per liter (μM) refer to the minimum concentration required to achieve 100% growth inhibition. MBC values (µM) refer to the minimum concentration required to kill 100% of the bacteria. Activities were measured in Zobell medium at 400 mM NaCl. All bacteria were isolated from *C. gigas* during mortality episodes. Under the test conditions, no activity was recorded against *Amphitrea* sp. 14.114-3T2, *Arcobacter* sp. 08.122-3T1, *Arcobacter* sp. 14.060-1T2, *Colwellia* sp. 09.021-3T1, *Idiomarina* sp. 15.5813, *Photobacterium* sp. 08.091-5T2, *Psychrobacter* sp. 15.5825, *Shewanella* sp. 15.5812, *Vibrio crassostreae* 7F5-29, *Vibrio harveyi* Th15-O-A01, *Vibrio tasmaniensis* 7G7-3. The synergies of *Cg*-BigDef1 and *Cg*-BigDef5 were measured as described previously [35]. Results are expressed as FIC index values according to the following formula: FIC = [*Cg*-BigDef1]/MIC_*Cg*-BigDef1_ + [*Cg*-BigDef5]/MIC_*Cg*-BigDef5_, where MIC_*Cg*-BigDef1_ and MIC_*Cg*-BigDef5_, are the MICs of the *Cg*-BigDef1 and *Cg*-BigDef5 tested alone and [*Cg*-BigDef1] and [*Cg*-BigDef1] are the MICs of the two peptides tested in combination. FIC index values are interpreted as follows: <0.5, strong synergy; 0.5 to 1, synergy; 1 to 2: additive effect; 2, no effect; >2, antagonism. *nt* stands for not tested. Boldface is used for active concentrations (MIC, MBC) and synergistic combinations of *Cg*-BigDefs (FIC).

## Data Availability

MALDI spectra, 16S sequences and amplicon sequences for microbiota analysis have been made available on the Ifremer Sextant catalog https://doi.org/10.12770/261d7864-a44c-43ab-b0c6-57fdaf7360ac (accessed on 14 October 2022); https://doi.org/10.12770/0d529567-92fd-4dcd-9d9c-70e98ab6f772 (accessed on 14 October 2022. Raw sequence data of 16S sequencing for Metabarcoding analysis are available on on the Ifremer Sextant catalog https://doi.org/10.12770/c960676e-2515-46f0-a313-4a91ac91908a (accessed on 17 October 2022).

## Supplementary Materials

The following supporting information can be downloaded at: https://www.mdpi.com/article/10.3390/md20120745/s1, Table S1: Permutational multivariate analysis of variance (PERMANOVA) table of oyster microbiota at ASV level for experimental anesthesia; Table S2: Permutational multivariate analysis of variance (PERMANOVA) table of oyster microbiota at ASV level comparing experimental injections (i.e. oysters injected with ASW/ with Cg-BigDef1); Table S3: Results of ad hoc pairwise PERMANOVA testing for differences in oyster microbiota at ASV level between experimental conditions (i.e. injected with ASW/ injected with Cg-BigDef1); Table S4: List of standard strains used as reference sequences in the 16S phylogenetic analysis; Figure S1: Species richness rarefaction curves; Figure S2: Lack of effect of anesthesia on the oyster microbiome; Figure S3: No changes in oyster microbiota α-diversity after injection of Cg-BigDef1; Figure S4: Differences in oyster microbiota between ASW and Cg-BigDef1 conditions for the top10 genera; Figure S5: ASVs differentially represented at T24 in oyster injected with CgBigDef-1 or ASW; Figure S6: No changes in total bacterial load in oysters injected with Cg-BigDef1; Figure S7: Chemical synthesis of Cg-BigDef5 used in this study.
