# Supplementary material for: Functional Diversification of Oyster Big Defensins Generates Antimicrobial Specificity and Synergy against Members of the Microbiota
Source: Mar Drugs. 2022 Nov 26;20(12):745. doi: 10.3390/md20120745 (PMC9786018; doi:10.3390/md20120745)
Supplement: Supplementary file 1 [file marinedrugs-20-00745-s001.zip › marinedrugs-2029059-supplementary.pdf]

Supplementary Materials

Functional Diversification of Oyster Big Defensins generates Antimicrobial Specificity and Synergy against members of the Microbiota

De San Nicolas N[1], Asokan A[2], Rosa RD[3], Voisin SN[4], Travers MA[1], Rocha G[3], Dantan L[1], Dorant Y[1], Mitta G[1,5], Petton B[6], Charrière GM[1], Escoubas JM[1], Boulo V[1], Pouzadoux J[1], Meudal H[2], Loth K[2,7], Aucagne V[2], Delmas AF[2], Bulet P[4,8], Montagnani C[1] and Destoumieux-Garzón D[1] *

[1] IHPE, Univ. Montpellier, CNRS, Ifremer, Univ. Perpignan Via Domitia, Montpellier, France
[2] Centre de Biophysique Moléculaire UPR4301 CNRS, Orléans, France
[3] Laboratory of Immunology Applied to Aquaculture, Department of Cell Biology, Embryology and Genetics, Federal University of Santa Catarina, Florianópolis, Santa Catarina, Brazil
[4] Plateforme BioPark d'Archamps, Archparc, Archamps, France
[5] Ifremer, IRD, ILM, Université de Polynésie Française, UMR EIO, Vairao, French Polynesia
[6] Ifremer, CNRS, IRD, Ifremer, LEMAR UMR 6539 Université de Bretagne Occidentale, Argenton-en-Landunvez F-29840, France
[7] UFR ST, Université d'Orléans, Orléans, France
[8] CR UGA, IAB, INSERM U1209, CNRS UMR5309, La Tronche -Archamps, France

*Correspondence: Delphine Destoumieux-Garzon

Tel.: +33 467144625

E-mail: Delphine.Destoumieux.Garzon@ifremer.fr

Table of Contents:

**Table S1.** Permutational multivariate analysis of variance (PERMANOVA) table of oyster microbiota at ASV level for experimental anesthesia.	Page 3
**Table S2.** Permutational multivariate analysis of variance (PERMANOVA) table of oyster microbiota at ASV level comparing experimental injections (i.e. oysters injected with ASW/ with Cg-BigDef1).	Page 3
**Table S3.** Results of ad-hoc pairwise PERMANOVA testing for differences in oyster microbiota at ASV level between experimental conditions (i.e. injected with ASW/ injected with Cg-BigDef1).	Page 3
**Table S4.** List of standard strains used as reference sequences in the 16S phylogenetic analysis.	Page 4
**Figure. S1.** Species richness rarefaction curves.	Page 6
**Figure S2.** Lack of effect of anesthesia on the oyster microbiome.	Page 7
**Figure S3.** No changes in oyster microbiota α-diversity after injection of Cg-BigDef1.	Page 8
**Figure S4.** Differences in oyster microbiota between ASW and Cg-BigDef1 conditions for the top10 genera.	Page 9
**Figure S5.** ASVs differentially represented at T24 in oyster injected with CgBigDef-1 or ASW.	Page 10
**Figure S6.** No changes in total bacterial load in oysters injected with Cg-BigDef1.	Page 11
**Figure S7.** Chemical synthesis of Cg-BigDef5 used in this study.	Page 12
**SM. References**	Page 13

Table S1. Permutational multivariate analysis of variance (PERMANOVA) table of oyster microbiota at ASV level for experimental anesthesia. Significance was obtained from n= 1,000 permutations.

Source of variation	DF	Sum of Squares	Mean Squares	F	Pvalue
Anesthesia	1	0.21	0.09	1.43	0.080
Residuals	14	2.05	0.90	-	-
Total	15	2.26	1	-	-

Table S2. Permutational multivariate analysis of variance (PERMANOVA) table of oyster microbiota at ASV level comparing experimental injections (*i.e.* oysters injected with ASW/ with *Cg*-BigDef1). Significance obtained from n= 1,000 permutations.

Source of variation	DF	Sum of Squares	Mean Squares	F	Pvalue
Injection	1	0.26	0.02	1.55	0.055
Time	2	2.58	0.23	7.43	0.001
Injection:Time	2	0.64	0.05	1.85	0.005
Residuals	42	7.29	0.67	-	-
Total	47	1.07	1	-	-

Table S3. Results of ad-hoc pairwise PERMANOVA testing for differences in oyster microbiota at ASV level between experimental conditions (i.e. injected with ASW/ injected with *Cg*-BigDef1). Statistical differences were tested for each kinetic time (i.e. T0, T6 and T24). Significance obtained from n= 1,000 permutations. Significant differences are bolded.

Source of variation	DF	Sum of Squares	R^2	F	Pvalue
T0 - injection	1	0.1722	0.057	0.849	0.754
T0 - Residual	14	2.8382	0.943	-	-
T0 - Total	15	3.0104	1.000	-	-
**T6 - injection**	**1**	**0.5169**	**0.143**	**2.331**	**$6.9e^{-4}$**
T6 - Residual	14	3.1039	0.857	-	-
T6 - Total	15	3.6208	1.000	-	-
**T24 - injection**	**1**	**0.2258**	**0.143**	**2.333**	**0.005**
T24 - Residual	14	1.3550	0.857	-	-
T24 - Total	15	1.5808	1.000	-	-

Table S4. List of standard strains used as reference sequences in the 16S phylogenetic analysis.

Genbank Accession	Taxon
CP003466	*Alcanivorax dieselolei*
CP008849	*Alteromonas australica*
CIP110805T	*Alteromonas mediterranea*
AY926460	*Alteromonas hispanica*
CIP103198T	*Alteromonas macleodii*
CP001999	*Arcobacter nitrofigilis*
FJ573217	*Arcobacter bivalviorum*
LCUH01000004	*Arcobacter porcinus*
ABQL01000001	*Bacillus subtilis*
KZ987423	*Bacillus fortis*
AF483625	*Bacillus aquimaris*
MACF01000036	*Bacillus mobilis*
AY651070	*Bizionia paragorgiae*
CP002534	*Cellulophaga lytica*
DQ007434	*Colwellia polaris*
JH393258	*Halomonas boliviensis*
JQ762286	*Halomonas zhaodongensis*
AM229314	*Halomonas gomseomensis*
AE017340	*Idiomarina loihiensis*
AJ440214	*Idiomarina baltica*
LGOW01000020	*Idiomarina abyssalis*
LTAV01000043	*Marinomonas aquimarina*
LTAW01000025	*Marinomonas atlantica*
LTAX01000041	*Marinomonas gallaica*
X67025	*Marinomonas vaga*
AQWH01000065	*Martelella mediterranea*
LELC01000058	*Photobacterium swingsii*
D25307	*Photobacterium angustum*
ADBS01000001	*Photobacterium damselae*
X67024	*Pseudoalteromonas haloplanktis*
AY563031	*Pseudoalteromonas marina*
CP013187	*Pseudoalteromonas phenolica*
CP011925	*Pseudoalteromonas piscicida*
LRRU01000009	*Pseudoalteromonas gelatinilytica*
X82140	*Pseudoalteromonas undina*
AB453700	*Psychrobacter piscatorii*
jgi.1058070	*Ruegeria marina*
LQBQ01000015	*Ruegeria marisrubri*
AP011177	*Shewanella violacea*
FR744784	*Shewanella electrodiphila*
AB081757	*Shewanella marinintestina*
MRZO01000018	*Staphylococcus xylosus*

ABID01000006	*Sulfitobacter indolifex*
jgi.1067976	*Sulfitobacter delicatus*
AM746476	*Tenacibaculum soleae*
RCCS01000002	*Tenacibaculum discolor*
KC261665	*Winogradskyella undariae*
U64013	*Winogradskyella rapida*
X74689	*Vibrio aestuarianus subsp aestuarianus*
CCJW01000022	*Vibrio crassostreae*
AJ514912	*Vibrio tasmaniensis*
BCUF01000119	*Vibrio harveyi*
CIP108868T	*Pseudoalteromonas spongiae*
AY563030	*Marinobacterium halophilum*
KT366925	*Marinobacterium aestuariivivens*

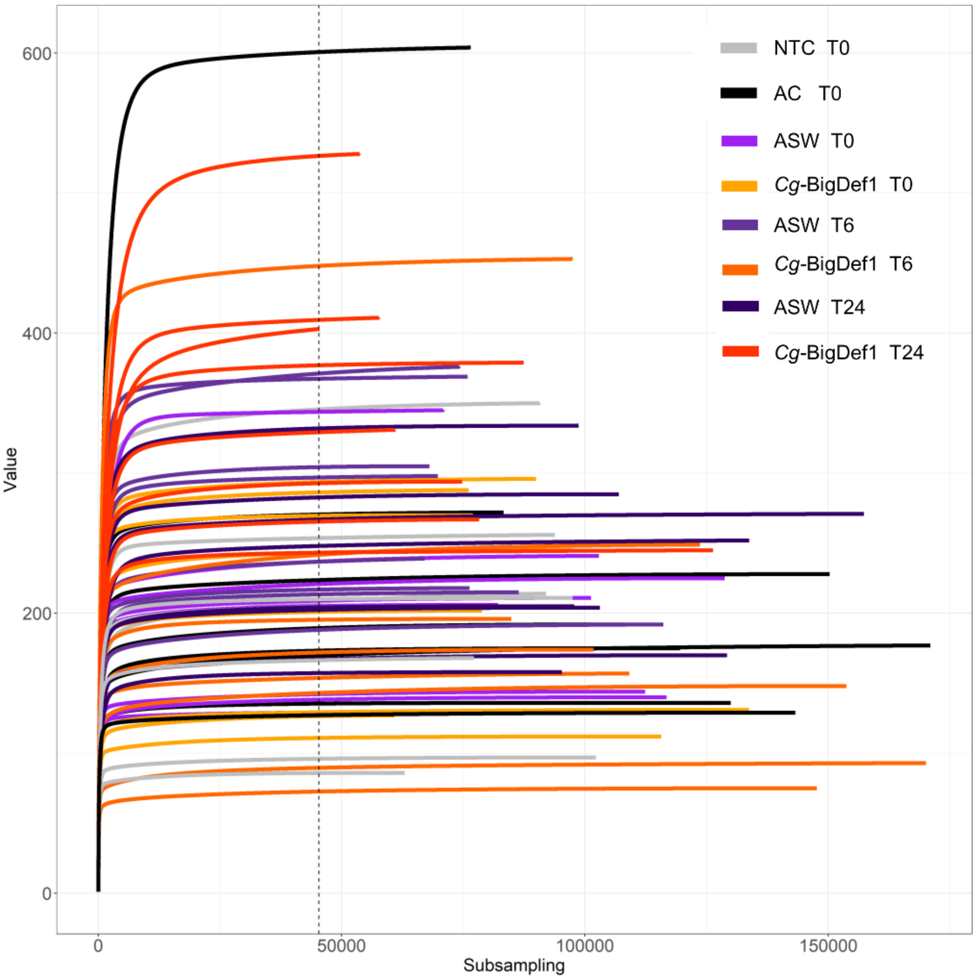

Figure S1. Species richness rarefaction curves.

Rarefaction curves are displayed for each sample (8 oysters per condition). Each color refers to a different condition. A subsampling was performed at 45.361 reads per sample (dashed line) for comparison of genera and ASV, as well as for β-diversity estimation.

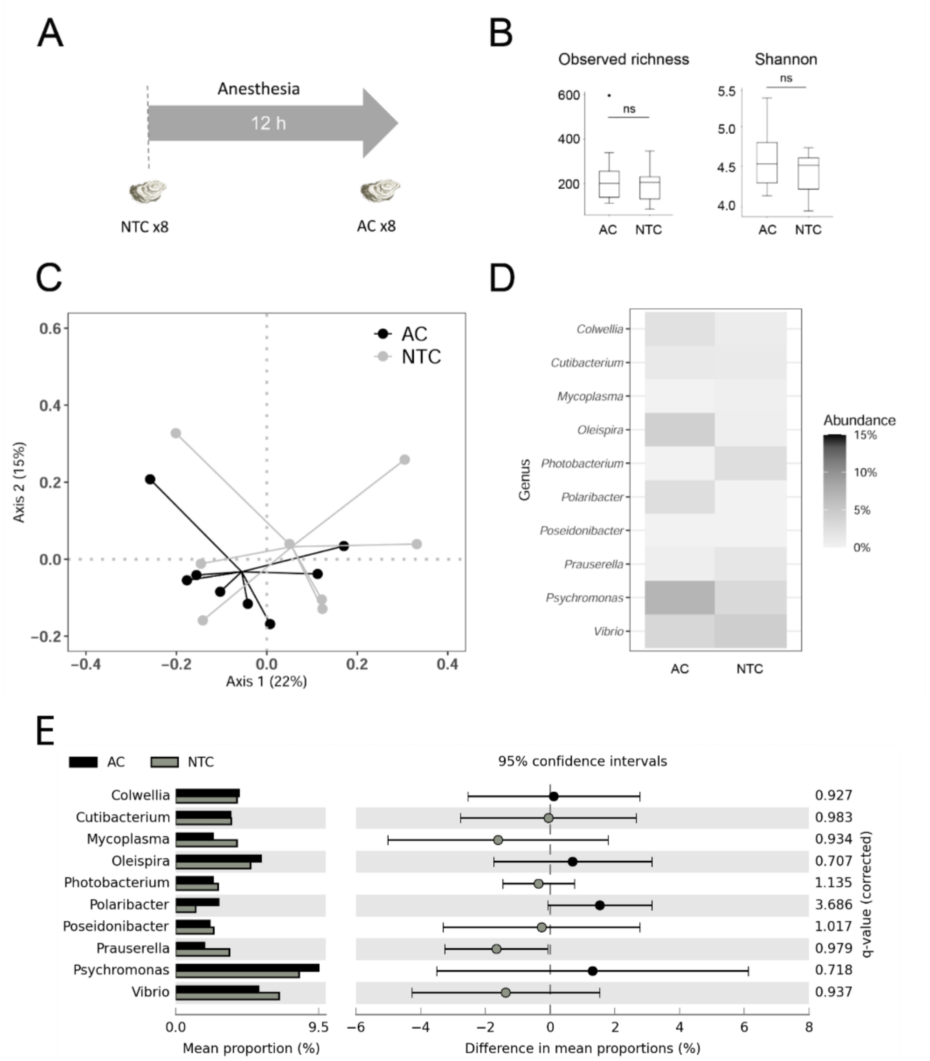

Figure S2. Lack of effect of anesthesia on the oyster microbiome.

A: Schematic representation of the experimental design used to test effect of anesthesia. From the same batch of oysters, 8 non-treated control oysters (NTC) and 8 anesthesia control oysters (AC) were collected. **B**: α-diversity of oyster microbiota, specific richness and Shannon indices (intraspecific diversity) in NTC and AC samples. **C**: β-diversity of oyster microbiota visualized by principal coordinate analysis (PCoA) biplot based on Bray-Curtis distances. Points colored in black and grey represents the microbiota for anesthetized oysters (AC) and non-treated oysters (NTC) respectively. **D**: Heatmap showing the relative abundance of the ten most common bacterial genera in AC and NTC samples. Each cell represents the mean proportion (based on relative abundance) carried by genera among samples associated with each condition considered (i.e. AC and NTC). **E**: Relative abundance of the ten most common genera observed in AC and NTC oysters**.** Results were analyzed by Welch's t-test with the Benjamini-Hochberg procedure, which controls the false discovery rate (FDR) using STAMPS software.

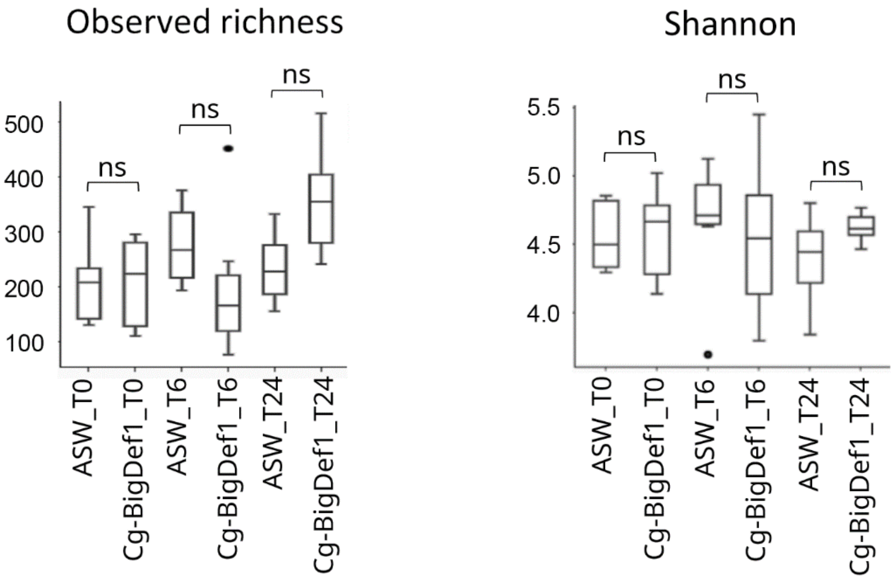

Figure S3. No changes in oyster microbiota α-diversity after injection of *Cg*-BigDef1.

α-diversity analyses of the oyster microbiota in 48 samples corresponding to oysters injected with *Cg*-BigDef1 or artificial seawater (ASW). Specific richness and Shannon (intraspecific diversity) indices were calculated in all samples. T0, T6 and T24 correspond to the time post-injection in hour.

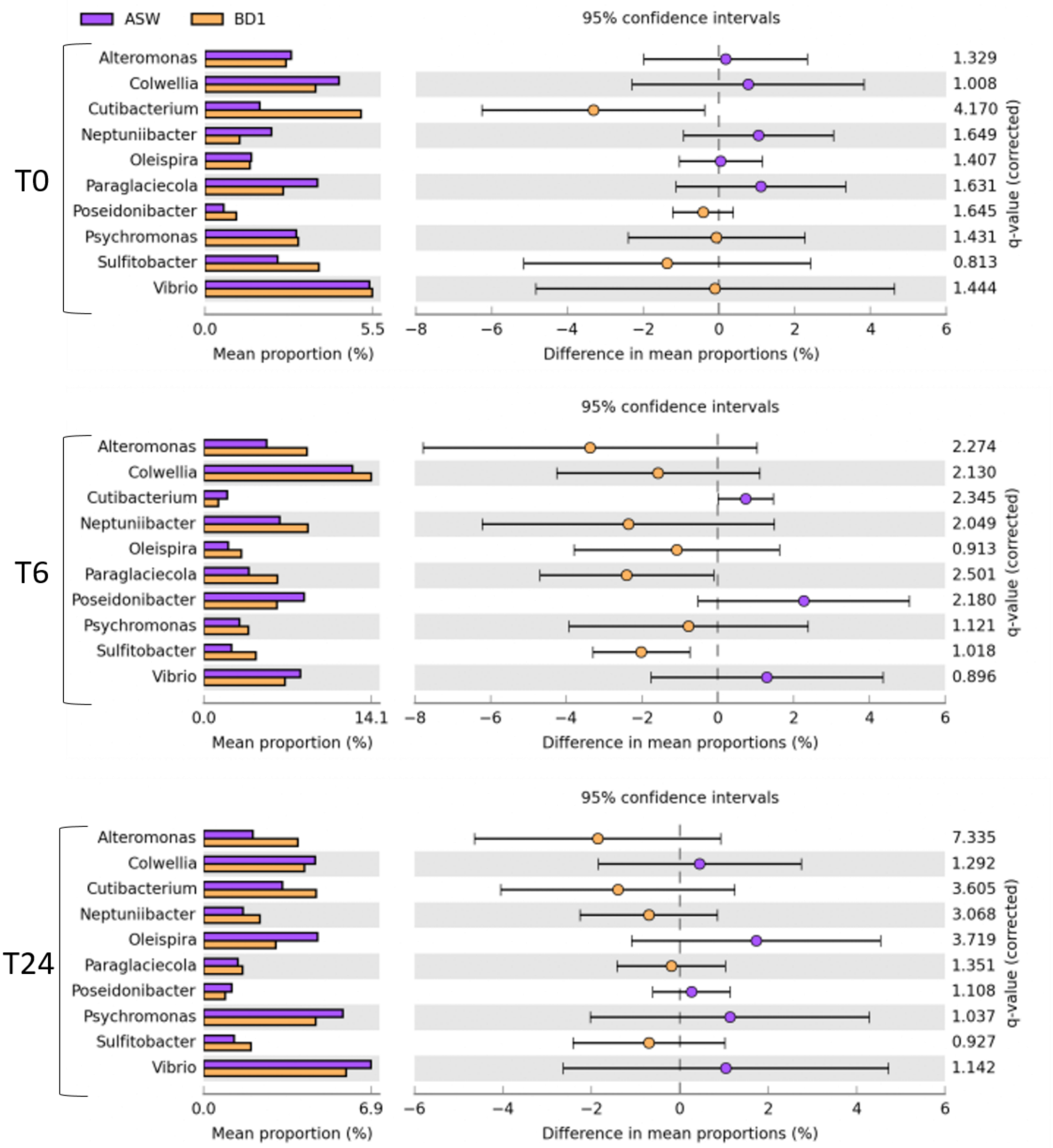

Figure S4. Differences in oyster microbiota between ASW and *Cg*-BigDef1 conditions for the top10 genera.

Relative abundance of the ten most common genera observed in ASW and *Cg*-BigDef1 injected oysters sampled at T0, T6 or T24**.** Results were analyzed by Welch's t-test using STAMPS software. The p-values were corrected with Benjamini–Hochberg FDR method.

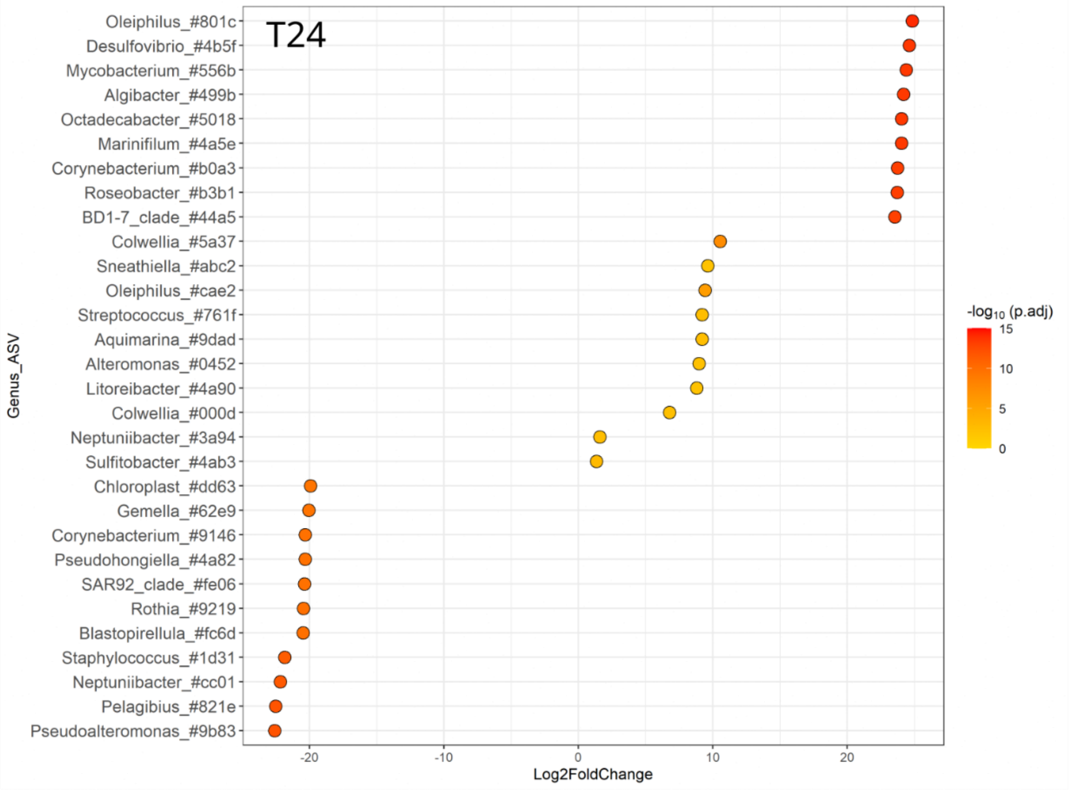

Figure S5. ASVs differentially represented at T24 in oyster injected with CgBigDef-1 or ASW.

Differential abundance analysis (DESeq2) at the ASV level between oysters injected with ASW and *Cg*-BigDef1 at T24. Each circle represents an ASV showing significant log2Foldchange (adjusted pvalue < 0.01) between experimental condition. Positive $\log_2$FoldChange means enrichment in *Cg*-BigDef1-injected oysters and negative $\log_2$FoldChange means enrichment in ASW-injected oysters. Taxa are denoted by their attributed genus followed by first four characters of the ASV barcode attributed by SAMBA. Note that ASVs without genera annotation were not represented in the figure. Differential abundance analysis identified 38 ASVs at T24 that were significantly enriched or impoverished in *Cg*-BigDef1-treated oysters. Among these, 30 ASVs were affiliated to 26 known genera.

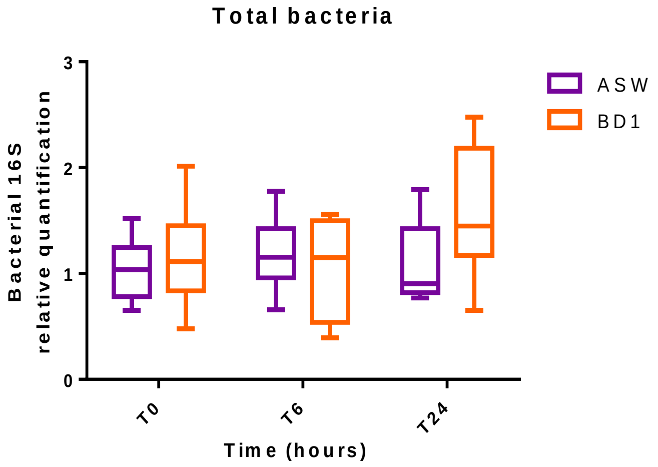

Figure S6. No changes in total bacterial load in oysters injected with *Cg*-BigDef1.

Relative quantification of total bacteria abundance were measured by qPCR. Boxplots represent the mean values and standard deviation for 8 oysters at various times (0, 6 and 24 h) post-injection with artificial sea water (purple) or *Cg*-BigDef1 (orange, 5µM final concentration). No significant differences were observed.

Chemical synthesis of *Cg*-BigDefs used in this study

Cg-BigDef1 was synthesized as already described in Terrier *et al.* [1] and Loth *et al*. [2]. Following a similar synthetic scheme, *Cg*-BigDef5 was obtained using a combination of solid phase chemical synthesis and native chemical ligation (NCL) followed by a step of oxidative folding under thermodynamical control (see Asokan manuscript in preparation for detail).

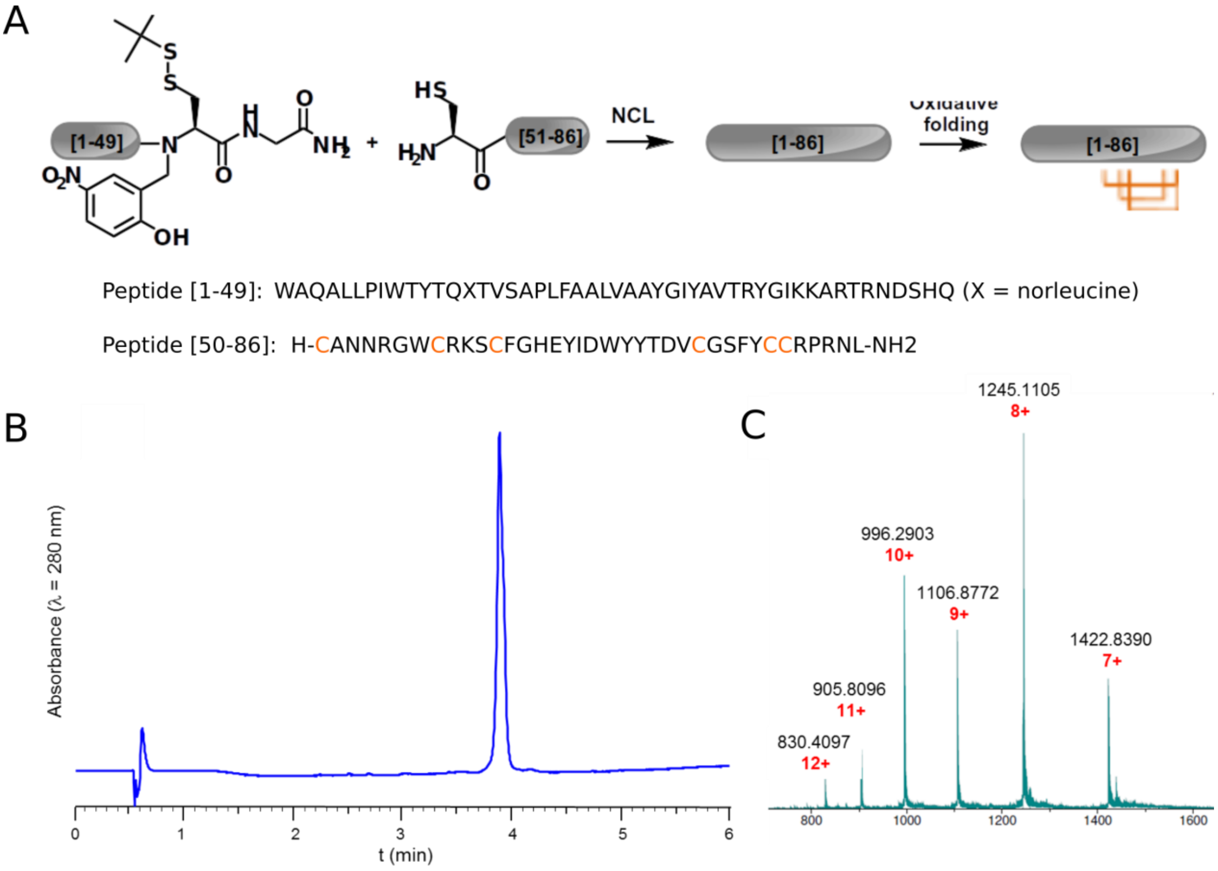

Figure S7. Chemical synthesis of *Cg*-BigDef5 used in this study

A. Synthetic scheme of *Cg*-BigDef5. **B.** High performance liquid chromatography (HPLC) analysis of *Cg*-BigDef5. t_R = 3.8 min, (Chromolith C18, 20-90% methanol gradient in $H_2$0 containing 0.1% TFA over 5 min). **C.** Electrospray ionization (ESI) - high resolution mass spectrometry (HRMS) of *Cg*-BigDef5. [M+H]+ calculated for C452H664N126O119S6: 9953.8178 Da, found: 9953.8343 Da (monoisotopic mass, deconvoluted) corresponding to a calculated molecular weight ([M]) of 9958.3 Da.

SM References

1. Terrier, V.P.; Adihou, H.; Arnould, M.; Delmas, A.F.; Aucagne, V. A Straightforward Method for Automated Fmoc-Based Synthesis of Bio-Inspired Peptide Crypto-Thioesters. *Chem. Sci.* **2015**, doi:10.1039/C5SC02630J.

2. Loth, K.; Vergnes, A.; Barreto, C.; Voisin, S.N.; Meudal, H.; Silva, D.; Bressan, A.; Bulet, P.; Touqui, L.; Delmas, A.F.; et al. The Ancestral N-Terminal Domain of Big Defensins Drives Bacterially Triggered Assembly into Antimicrobial Nanonets Karine. *MBio* **2019**, 10:e01821-19, doi:10.1128/mBio.01821-19.
